# Effects of Carrageenans on Biological Properties of Echinochrome

**DOI:** 10.3390/md16110419

**Published:** 2018-11-01

**Authors:** Ekaterina V. Sokolova, Natalia I. Menzorova, Victoria N. Davydova, Alexandra S. Kuz’mich, Anna O. Kravchenko, Natalya P. Mishchenko, Irina M. Yermak

**Affiliations:** G.B. Elyakov Pacific Institute of Bioorganic Chemistry, Far-East Branch of the Russian Academy of Sciences, Prospect 100-let Vladivostoku, 159, 690022 Vladivostok, Russia; menzor@piboc.dvo.ru (N.I.M.); vikdavidova@yandex.ru (V.N.D.); assavina@mail.ru (A.S.K.); Kravchenko_89@mail.ru (A.O.K.); mischenkonp@mail.ru (N.P.M.); imyer@mail.ru (I.M.Y.)

**Keywords:** carrageenan, algae, echinochrome, reactive oxygen species, cytokines, HT-29

## Abstract

Sea urchin pigment echinochrome A (Ech), a water-insoluble compound, is the active substance in the cardioprotective and antioxidant drug Histochrome^®^ (PIBOC FEB RAS, Moscow, Russia). It has been established that Ech dissolves in aqueous solutions of carrageenans (CRGs). Herein, we describe the effects of different types of CRGs on some properties of Ech. Our results showed that CRGs significantly decreased the spermotoxicity of Ech, against the sea urchin *S. intermedius* sperm. Ech, as well as its complex with CRG, did not affect the division and development of early embryos of the sea urchin. Ech reduced reactive oxygen species production (ROS) in neutrophils, caused by CRG. The obtained complexes of these substances with pro- and anti-activating ROS formation properties illustrate the possibility of modulating the ROS induction, using these compounds. The CRGs stimulate the induction of anti-inflammatory IL-10 synthesis, whereas Ech inhibits this synthesis and increases the production of the pro-inflammatory cytokines IL-6 and TNFα. The inclusion of Ech, in the complex with the CRGs, decreases Ech’s ability to induce the expression of pro-inflammatory cytokines, especially TNFα, and increases the induction of anti-inflammatory cytokine IL-10. Thus, CRGs modify the action of Ech, by decreasing its pro-inflammatory effect. Whereas, the Ech’s protective action towards human epithelial HT-29 cells remains to be unaltered in the complex, with κ/β-CRG, under stress conditions.

## 1. Introduction

With increasing awareness of functional properties of products from marine organisms, their attractiveness, both as a source of nutritious food items and stockpot of novel, biologically-active compounds, continuously expand [1]. The characteristic color of spines and armors of sea urchins are attributed to calcium salts of polyhydroxynaphthoquinone pigments derivatives—spinochromes and echinochromes—exhibiting a vast range of pharmacological activities. One of the most popular pigments is the echinochrome A (6-ethyl-2,3,5,7,8-pentahydroxy-1,4-naphthoquinone), which is known to be a biologically-active compound with antimicrobial, antialgal, and antioxidant activities [2]. Most of all, it has been ascertained that a treatment with echinochrome protected the mitochondrial functions in cardiomyocytes, against the acardiotoxic drugs (*tert*-Butyl hydroperoxide, sodium nitroprusside) [3]. There are controversial data in the literature, in terms of Ech’s capacity to activate an immune response, some data suggest that Ech has an ability to activate inflammation, whereas others suggest that it can suppress inflammation [4,5]. Pharmacological studies in vitro and in vivo demonstrated that naphthoquinone pigments have a wide therapeutic latitude and are nontoxic, at therapeutic doses [6]. 

In Russia, echinochrome is produced from the sand dollar *Scaphechinus mirabilis*. It is the active substance in the cardioprotective and antioxidant drug Histochrome^®^ (C (Ech) = 0.2 mg mL^−1^) and is available in ampoules, permitted for subconjunctival, parabulbar, or intravenous administration [7,8]. One of the main setbacks to the wide use of Ech, is its insolubility in aqueous solutions and high susceptibility to oxidative destruction. Improvement of therapeutic efficacy of drugs can be achieved by modifying the formulation technique, for instance, by means of polymeric systems. Marine natural edible polymers have been widely used in hydrogels, drug encapsulation, and drug delivery because of their benefits, comprising such advantages as biocompatibility, biodegradability, and adhesiveness [9,10]. 

In order to provide a stable and biocompatible environment to the Ech, polymeric matrix systems, based on polysaccharides from red algae, have been proposed to be suitable candidates for oral delivery [11]. Polysaccharides of red algae, carrageenans (CRGs) are a class of linear galactans with alternating 1,3- and 1,4-linked galactose residues (d- and G-units). Several types of these polysaccharides were identified, based on the structure of their disaccharide repeating units, the pattern of sulfation, and the presence of 3,6-anhydrogalactose (DA-unit), as a 4-linked residue [12]. The three most industrially-exploited types, in the order of increasing sulfation degrees and decreasing gelation capabilities, respectively, are the κ-, ι- and λ- CRGs. Natural CRGs are often hybrids of more than one of these units and are composed of several carrabiose moieties, the proportions and structures of which vary with species, the life stages of seaweeds, and the ecophysiological and developmental conditions [13,14,15]. CRGs are widely utilized due to their excellent physical properties, such as thickening, gelling, and stabilizing effects in the food industry [16,17]. CRGs have successfully become appealing tools in immunotherapy and drug delivery, due to their immuno-active features and valuable physical properties as gelling [18,19].

Recently we have established that Ech is incorporated into the CRG supramolecular structure, which results in formation of complexes with altered Ech properties, such as decreased oxidative degradation and improved solubility. Along with the suitable physico-chemical properties, an Ech complex with CRG, in mice, revealed a high gastroprotective activity, surpassing the effect of either of the components used alone [11].

Unravelling the influence of CRGs–Ech complexes on some immunological parameters (ROS formation in phagocytic cells, the cytokine production in human whole blood model) and on human epithelial cell monolayers, will provide essential information for the rational design of CRG-based matrices for Ech delivery, for oral administration. It would also inspire the design of new approaches in assessing the modification of biological properties of biomaterials, used in delivery systems. The aim of this work was to investigate the biological properties of Ech that was included in a CRG matrix.

## 2. Results

CRGs were isolated by aqueous extraction from the non-fruited form of red algae *Chondrus armatus* (Gigartinaceae), *Tichocarpus crinitus* (Tichocarpaceae), and *Ahnfeltiopsis flabelliformis* (Phyllophoraceae), harvested along the Russian coast of the Japanese Sea and separated using 4% KCl into the KCl-insoluble and KCl-soluble fractions. The structures of polysaccharides were studied by ^13^C-NMR and FT-IR-spectroscopy. The obtained spectra have been compared with the spectra of polysaccharides, which we had isolated earlier, from the above-mentioned species of algae [20,21,22]. The identity of the spectra indicates that the KCl-insoluble fraction form of *C. armatus*, *T. crinitus*, *A. flabelliformis*, were κ-, κ/β-, and ι/κ-types, respectively, and a KCl-soluble fraction from was *C. armatus*–λ-CRG.

The structures of disaccharide repeating units of the carrageenans and the sulfate content, as well as the average molecular weights of all the samples investigated, are summarized in Table 1. CRGs differ from each other in number and position of sulfated groups, and in the presence (κ, κ/β and ι/κ) or absence (λ) of 3,6-anhydrogalactose units (DA). The degree of sulfation decreased in the following row: λ > ι/κ > κ > κ/β.

### 2.1. Toxicity

As a cellular model to study spermotoxic and embryotoxic properties, we used the spermatozoa and developing embryos of the sea urchin *S. intermedius*. 

**Figure 1:**
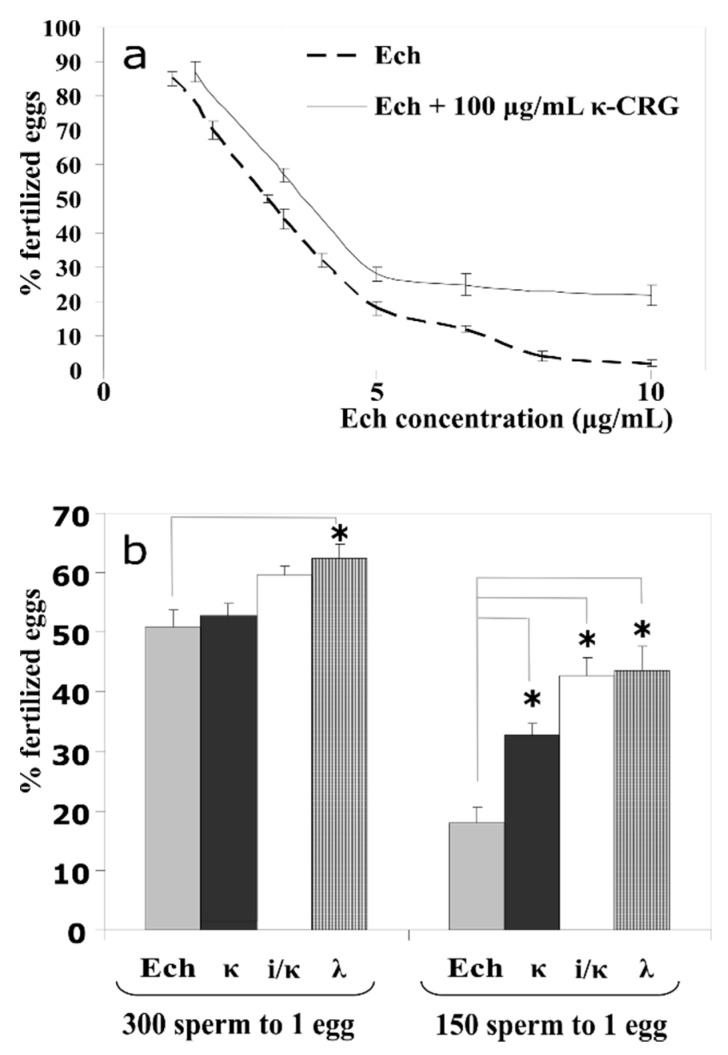
(**a**) The influence of Echinochrome (Ech) and its complex with the κ-carrageenans (CRGs) (100 μg mL^−1^), on the sea urchin spermatozoa fertilizing ability (spermatozoa to eggs ratio 300:1). (**b**) The spermatozoa fertilizing ability of various types of CRGs (100 μg mL^−1^), in the presence of Ech (3 mg mL^−1^). * *p* < 0.05.

The spermotoxicities of Ech, CRGs, and the complexes Ech/CRGs were investigated by the Sea Urchins Sperm Cell Toxicity Test (SUSCT)-test [23,24]. These activities were determined by the degree of inhibition of the spermatozoa’s ability to fertilize the sea urchin eggs. Previously, all types of CRGs (κ, λ, ι/κ) were investigated for spermotoxicity in seawater, at a concentration 50 to 200 μg mL^−1^. The results showed that all of the studied CRGs had no toxic effect on the spermatozoa fertilization ability, at these concentrations. The current study with Ech in the concentration range from 1 to 10 μg mL^−1^ revealed that this substance exhibited spermotoxicity. The spermotoxicity of Ech was expressed in the 50% inhibition of the spermatozoa’s ability to fertilize the egg-cells (IC_50_ values were of 3 μg mL^−1^), at a sperm:egg ratio of 300:1. When the Ech was added to a solution of CRGs, with a concentration of the 100 μg mL^−1^, the spermotoxicity of the Ech decreased significantly. The higher the concentration of the Ech, the greater was the protective effect of the CRG (Figure 1a). The protection of various CRGs types, against the spermotoxicity of the Ech (C = 3 μg mL^−1^) was studied by the SUSCT-test, at the spermatozoa to eggs ratios of 300:1 and 150:1 (Figure 1b). From the data presented in Figure 1b, it can be seen that λ-CRG, with a higher degree of sulfation, showed a greater protective activity. This dependence of the protective effects of the CRGs on their structures, was particularly noticeable at the spermatozoa to eggs ratio of 150:1, when the sensitivity of the method was the highest.

To determine the embryotoxic effects of the Ech and its complex, with the CRG (100 μg mL^−1^), fertilized eggs from the sea urchins were used. In a concentration range from 2–36 μg mL^−1^, the Ech did not affect the division and development of early embryos of the sea urchin *Strongylocentrotus intermedius*, as well as its complex with the CRGs.

### 2.2. Reactive Oxygen Species (ROS)-Inducing Activity of the CRG and the Ech on the Human Blood Cells

The ROS induction in human neutrophils, in the presence of the Ech and its complexes with CRGs, was determined with the Ech concentration varying from 1 to 10 μg mL^−1^. To assess the effect of the content of the polysaccharide in the complex with the Ech, on the formation of ROS, we used CRG concentrations in the range of 5 to 200 μg mL^−1^. Thus, complexes with different CRGs/Ech ratios (5:1, 10:1, and 20:1) were prepared.

**Figure 2:**
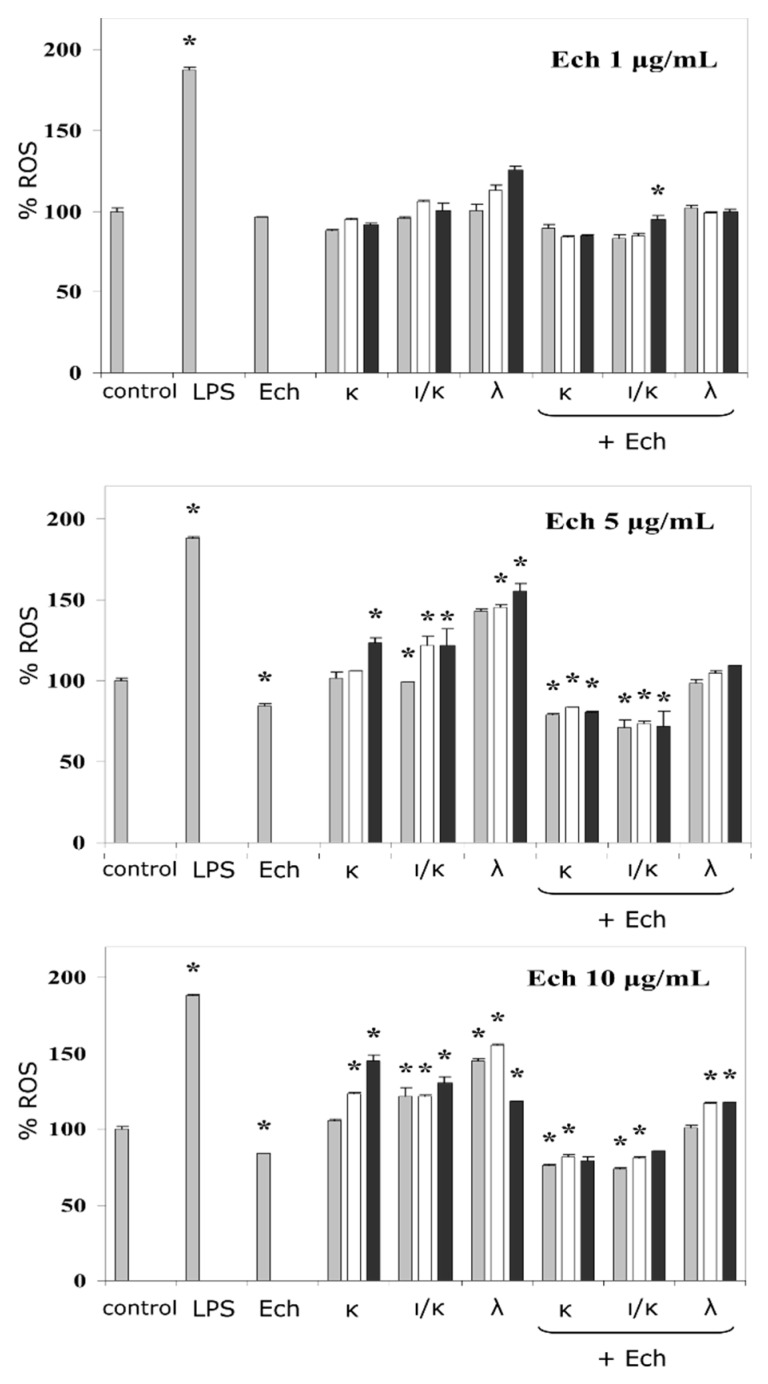
Neutrophils reactive oxygen species (ROS) formation in the presence of the Ech, CRGs, and their complexes. The concentration of the lipopolysaccharide (LPS) was 10 μg mL^−1^ and of the Ech was 1.0, 5.0, or 10.0 μg mL^−1^, final value. The concentrations of the CRGs alone and in complexes with the Ech changed in the following ratios row: 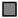 = 5:1; 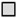 = 10:1; 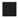 = 20:1. The results are expressed as % change in ROS, relative to the control (100%), * *p* < 0.05.

The corresponding effects were detected using a fluorescent probe and measured by means of flow cytometry (Figure 2). Lipopolysaccharide (LPS) from *E. coli* was used as a reference immunomodulator, in the current test, and the ROS production induced by the LPS, as a positive control, was approximately twice as much as the negative control (the vehicle). At low concentrations, the activity of Ech towards the ROS formation was comparable to the negative control, whereas at high concentrations its effect was lower than that of the control by 20%. The influence of the CRGs on the activation of ROS, at lower concentrations, was not significant except for the λ-CRG. In contrast, the CRGs at a concentration of 100 μg mL^−1^ intensified the induction of the ROS by up to 25–55%, relative to the negative control. The addition of the Ech to the CRGs, especially at high concentrations, resulted in significant diminishment of the ROS formation induced by the CRGs alone. The action of the complexes was compared to the negative control, where at higher concentrations, the effect of the samples was more noticeable.

### 2.3. IL-10-Inducing Action of the CRGs and the Ech on the Human Blood Cells

**Figure 3:**
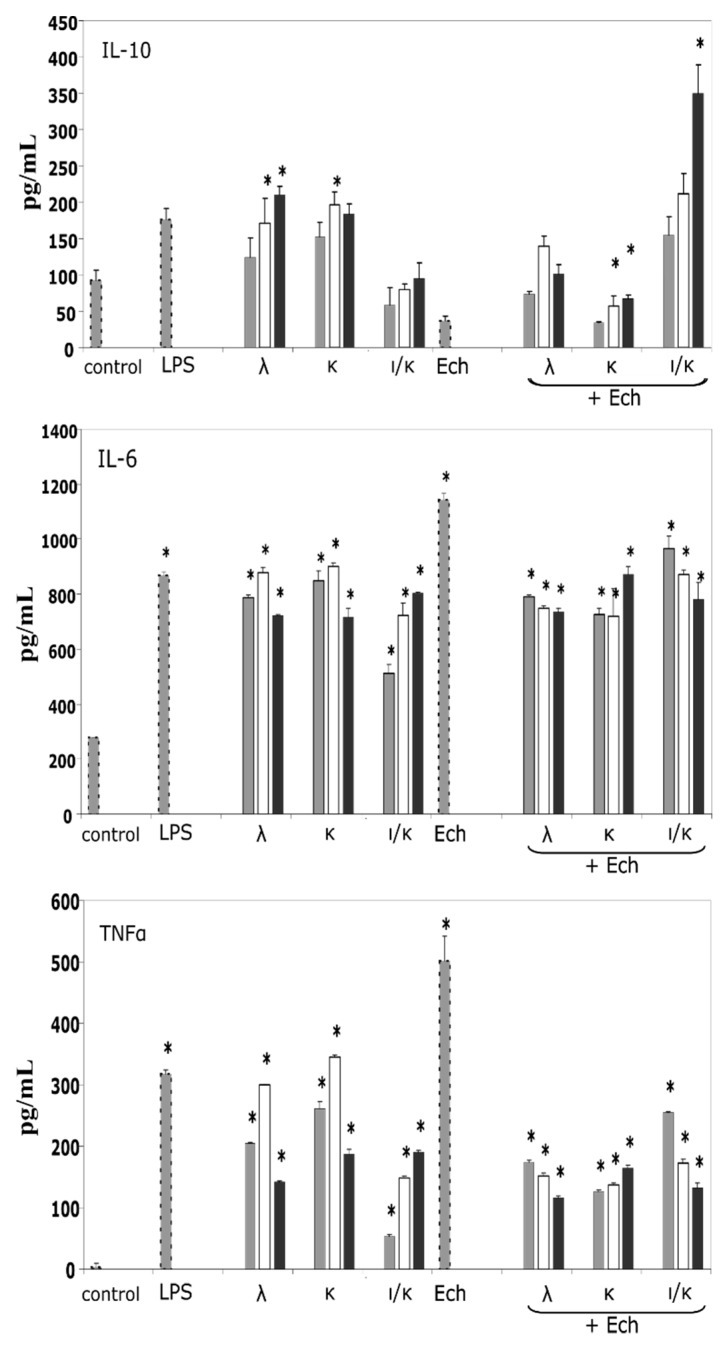
The induction of the necrosis factor-α, IL-6, and IL-10, in the presence of λ-, κ-, ι/κ-CRGs, Ech, and their complexes. The concentration of the LPS was 0.01 μg mL^−1^ and that of Ech was 1 μg mL^−1^, which were the final value. The concentrations of the CRGs, alone and in complexes with the Ech (1 μg mL^−1^), changed in the following rows: 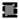 = control (saline/1 μg mL^−1^ of Ech); 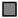 = 5 μg mL^−1^, final value; 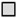 = 10 μg mL^−1^, final value; 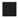 = 20 μg mL^−1^, final value. * *p* < 0.05.

The action of the carragenans, Ech, and their complexes on the pro-inflammatory (IL-6 and TNFα) and anti-inflammatory (IL-10) cytokines induction was conducted. In this experiment, Ech was used at one concentration, 1 μg mL^−1^ whereas, CRG concentrations individually and in complexes, varied in the following row 5.0, 10.0, and 20 μg mL^−1^. As seen in Figure 3, κ- and λ-types (10 and 20 μg mL^−1^) induced the expression of IL-10 in cells, by approximately 120 and 100 pg mL^−1^, in comparison to the negative control, respectively. Ech significantly inhibited the synthesis of IL-10, reducing the induction of this anti-inflammatory cytokine by 50%, compared to the control. At the same time, the inclusion of Ech into the CRG complex increased the induction of IL-10 synthesis, compared to Ech. The greatest effect was shown by the complex of Ech with ι/κ-CRG (Figure 3). Regarding the pro-inflammatory cytokines, Ech (1 μg mL^−1^) was a strong inductor, in comparison to the highest concentrations of the CRGs, but its action was decreased, especially in the complexes with the κ- or λ-types, by about 300 pg mL^−1^ for IL-6 and 350 pg mL^−1^ for TNFα. However, the combined action of the CRNs and the Ech complexes on the IL-6 still remained high, compared to the control. The ι/κ-CRG influenced the effect of Ech with less degree than the others, as complexes had formed. Thus, the CRGs modified the activity of Ech by decreasing its pro-inflammatory effect.

### 2.4. Influence of the CRGs, the Ech and Their Complex, on the HT-29 Tumor Cells

**Figure 4:**
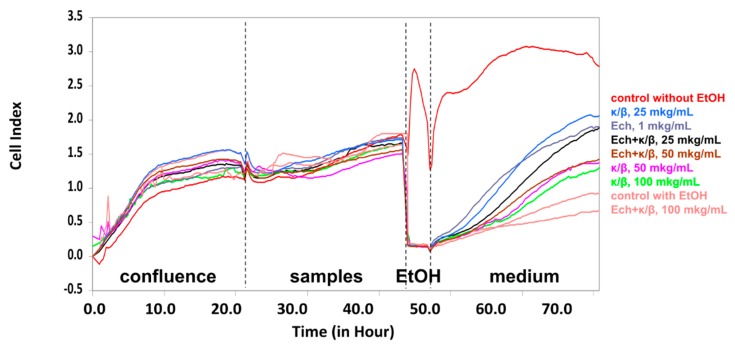
Time and dose-dependent cellular response profiling of HT-29 intestinal epithelial cells, in the presence of the κ/β-carrageenan, Ech, and their complex. Representative data are averaged from five wells. All experiments were repeated at least two times. Four stages of the experimental design are indicated with dashed lines: **1**—Growth of HT-29 cells to confluence; **2**—the stage of samples addition; **3**—incubation with ethanol; **4**—after the ethanol exposure. Concentration of the Ech (1 μg mL^−1^, final value) was fixed. After each stage, the culture medium (McCoy’s 5A Modified) was refreshed.

The effect of the Ech, alone and in carrageenans complexes, on the HT-29 cells treated with ethanol was investigated. The exposure of cells to EtOH permits an assessment of the samples’ ability to affect cell viability and, as a result, the permeability of the epithelial monolayer. All of the investigated samples were inert, in response to the intestinal epithelial HT-29 cells, under normal conditions. Under stress conditions, only the κ/β-CRG and the Ech, as well their complex, restored the cell viability after exposure to the EtOH. As to the CRG, the most prominent action was detected for the lowest concentrations, where protective effect preserved. The complex of κ/β-CRG with the Ech, also possessed an ability to restore the HT-29 cells after an exposure to ethanol (Figure 4).

## 3. Discussion

Ech, a water-insoluble compound, is the active substance (P N002362/01) of the drug Histochrome^®^, registered in the Russian Federation. Earlier we have shown that Ech is soluble in aqueous solutions of CRGs, up to the concentration of 0.1 mg mL^−1^. Moreover, the CRG environment protects the Ech from autooxidation [11]. In this work, we showed that carrageenans modified the biological activity of the Ech.

One of the manifestations of the biological effect of the drug is its ability to cause some disorders in the development and death of embryos (spermotoxic, embryotoxic, and cytostatic activities). The widespread use of the sea urchin embryos to test the toxicological and pharmacological effects of various drugs is due to the simplicity of the incubation of the synchronously developing embryos, under controlled conditions, and the ease of the intravital observation. The influence of the Ech and its complex with CRG, on sperm, was determined by the degree to which it inhibited the ability of spermatozoa to fertilize the sea urchin eggs and the further development of the early embryos of sea urchins, in comparison to the control. As the results showed, the CRGs significantly decreased the spermotoxicity of the Ech, towards the sea urchin *S. intermedius* sperm. Furthermore, neither the Ech, nor its complex with CRG, affected the division and development of the early embryos of the sea urchin.

Oxidative processes occurring during the neutrophils activation could be traced by the change in the ROS production [25]. In the case of the phagocytosis of the pathogens, the ROS were produced by the nicotinamide adenine dinucleotide phosphate oxidase (NOX) in a small volume of the phagosome [26]. During our study, we used an APF fluorescent probe (2-[6-(4-amino)phenoxy-3H-xanthen-3-on-9]benzoic acid), with a strong specificity towards the species of the reactive oxygen, localized predominantly in the phagosomes [27]. It should be noted that, in general, the activating effect of the CRGs was dependent on the polysaccharide concentration and the sulfation degree. In this study, a positive correlation between the impact on the ROS formation and the sulfation degree of the CRG (except for its highest concentration) was observed. Generally, the complex of the λ-CRG, with the Ech, was the most active out of all three types, their effect (complexes of the CRGs and the Ech with concentrations of 200:5 and 100:5 μg mL^−1^) was about 15%, compared to control (Figure 2). The importance of the CRG sulfation degree, with regards to monocyte behaviour, have also been observed, previously [28].

In the complexes of the Ech with polysaccharides, containing 3,6-anhydrogalactose and the lower sulfate group contents (κ, ι/κ), the resultant action was closer to the level of the Ech alone. This was supported by results from the literature, which reported that the Ech significantly prevented an increase in the ROS levels in rat cardiac myoblast H9c2 cells and cardiomyocytes induced by some cardiotoxic agents [3], as well as in intraocular inflammation caused by endotoxin-induced uveitis [29]. Overall, this experiment indicated the modulation of the inductions of the ROS, in complexes with substances containing pro- (CRGs) and anti- (Ech) activating properties.

Depending on the ROS location in cells, the function of these molecules changes enormously. For example, mitochondrial ROS have a particularly interesting role in the immune response, since these ROS are currently considered essential for pathways initiating the production of pro-inflammatory cytokines [30]. The influence of the investigated samples on the synthesis of the immune mediators enabled the study of another facet of their immune activity, both separately and as complexes. Pro-inflammatory cytokines were exemplified by the IL-6, the most important inducer of the acute-phase proteins, and the TNFα, another pro-inflammatory molecule with cytotoxic effects in antitumor immunity, whereas the IL-10 is an important immunoregulatory cytokine with multiple biologic effects and strong tendencies of anti-inflammatory action [31]. The CRGs stimulated the induction of anti-inflammatory IL-10, whereas, the Ech inhibited the synthesis of this cytokine, and the addition of the CRGs to the Ech increased the induction of the expression of the anti-inflammatory IL-10 (Figure 3). Ech, at a concentration 1 µg mL^−1^, increased the IL-6 and TNFα synthesis; however, the complexes with CRGs exhibited much less activity in the case of synthesizing the pro-inflammatory cytokine TNFα. The effect of the Ech on the cytokine balance towards the pro-inflammatory response corresponded to the literature data, which reported that spinochromes act as inductors of TNF-α production in LPS-stimulated macrophage cell cultures [5]. Another study underlined a pro-inflammatory action of the naphthoquinones, in mice [32].

Literature data suggest that the CRGs do not affect the epithelial cells of human gastrointestinal tract [33], but the influence of the Ech towards these cells, which is of special interest when one considers an oral administration of a drug, has not been investigated, to our knowledge. 

HT-29 is a colorectal cancer cell line used as an in vitro model, for the intestinal epithelium, because it is a mucin secreting cell line which retains many features attributed to the lower small intestine [34]. Previously we have studied the influence of CRGs on these cells, under stress conditions and have found out that only the low-sulfated CRG had a protective action towards the HT-29 intestinal epithelial cells [35]. Our purpose in the study described in this report, was to determine the protective action of the Ech alone and in combination with the low-sulfated CRG on the survival of monolayers of these cells, treated with EtOH (Figure 4). The stress effect of ethanol on the state of the HT-29 cells provided an opportunity to assay the protective properties of polysaccharides from the red algae and the Ech. The Ech (1 μg mL^−1^) also preserved the HT-29 cells, under stress conditions, to an extent similar to the κ/β-CRG (25 μg mL^−1^). These results provide an opportunity to propose the CRGs as a possible matrix system, for oral delivery of Ech, which preserves the Ech-favorable qualities and mitigates its negative biological properties.

In general, the CRGs modified the Ech toxicity and the immunological properties. Our results showed that the CRGs significantly decreased the spermotoxicity of the Ech, against the sea urchin *S. intermedius* sperm. The Ech, as well as its complex with CRG, did not affect the division and development of the early embryos of the sea urchin. The influence of the investigated substances on the induction of the ROS, in the neutrophils, confirmed that Ech in a complex with a polysaccharide inhibited the induction of ROS induced by CRG.

The complexes obtained by us illustrated the modulation of the ROS induction, by these substances, with pro- (CRGs) and anti- (Ech) activating properties of the initial components. The CRG decreased the Ech’s ability to induce the expression of pro-inflammatory cytokines and increased the expression of anti-inflammatory cytokines. Whereas, the Ech’s protective action towards the intestinal cells exposed to EtOH, remained invariable in the complex with the κ/β-CRG.

## 4. Materials and Methods 

The standardized echinochrome (pentahydroxyethylnaphthoquinone, Ech), registration number in the Russian Federation was P N002362/01 [Russian State Register of Drugs (as of 5 December 2016) Part 2]. It was obtained in powder form, from the G.B. Elyakov Pacific Institute of Bioorganic Chemistry, Vladivostok. The purity of the Ech (99.0%) was confirmed by liquid chromatography, coupled with mass spectrometry (LC-MS) data (Shimadzu LCMS-2020, Kyoto, Japan). The purified Ech that looked like red-brown needles, was soluble in ethanol, had a melting point of 219–221.5 °C, and a similar nuclear magnetic resonance (NMR) spectra to that reported previously in Reference [36]. We used an ethanolic solution of the Ech, at a concentration 10 mg mL^−1^, as a stock solution.

The CRGs were isolated by aqueous extraction from the *Chondrus armatus* (Gigartinaceae), *Tichocarpus crinitus* (Tichocarpaceae), and *Anfeltiopsis flabelliformis* (Phyllophoraceae) red algae, harvested along the Russian coast of the Japanese Sea. The polysaccharides were separated into gelling KCl-insoluble and non-gelling KC1-soluble fractions and their structures were established according to the published protocols [20,21,22]. Viscosimetric molecular weights of the CRGs were calculated using the Mark-Houwink equation: [η] = KMα, where [η] is the intrinsic viscosity and K and α are empirical constants constituting 3 × 10^−3^ and 0.95 at 25 °C in 0.1 M NaCl, for the CRGs. The commercial LPS was from the bacterium *Escherichia coli* 055:B5 (Catalog No. L2880, Lot No. 102M4017V, Sigma, St. Louis, MO, USA). An APF probe (2-[6-(4-amino)phenoxy-3H-xanthen-3-on-9]benzoic acid) was purchased from Assay Designs (cat No 906-043).

### 4.1. Sea Urchin Models

The test samples in these experiments were polysaccharides of three CRG types. They were dissolved in sea water at 50 °C, to the level of the initial concentrations, from 0.5 to 1.0 mg mL^−1^. Ech dissolved in 50% EtOH was used at the initial concentrations of 0.5 and 1 mg mL^−1^. Adult sea urchins *Strongylocentrotus intermedius* (collected in the Troitsa Bay (Peter the Great Bay, the Sea of Japan) during August–September 2017, at a depth of 5–10 m) were stored in an aquarium with a closed-filter system, at a seawater temperature of 20 °C and salinity of 32 ± 0.5‰. Pooling male and female gametes and eggs, fertilizations were performed according to the standard procedures described in References [37,38]. The quality of the isolated sperms and eggs was checked with fertilization, prior to experiment. The fertilization membrane was formed within 1–2 min, after insemination, in at least 95–99% of the eggs, under normal conditions.

### 4.2. Sea Urchins Sperm Cell Toxicity Test (SUSCT Test)

We used the standard bioassay record of the SUSCT test for the analysis of the obtained preparations [23,24]. Sperm from sea urchins (15 × 10^6^ cell mL^−1^), prior to the experiment, were sustained in seawater for 30 min, with various concentrations of the substances. Next, the spermatozoa were added to the suspension of eggs (2.5 × 10^3^ cell mL^−1^). The final sperm:egg ratios were about 300 and 150:1. After 15 min, the percentage of fertilized eggs were counted on a Motic AE 21 inverted microscope (Xiamen, China). The experiments were carried out in triplicate using a 12-well plate. The effect of substance toxicity was assessed, visually, according to the number of unfertilized eggs in the four fields of view, in each experiment. The number of eggs fertilized by the sperms, after incubation in the seawater, without substances (control), was assumed to be 95–100%.

### 4.3. Sea Urchin Embryos Development Test

The preliminary fertilized eggs (2.5 × 10^3^ cell mL^−1^) were stored in seawater, with different concentrations of the tested Ech (10 to 40 µg mL^−1^) and its complexes with CRGs, for 30 min, after which a suspension of native sperm was added. The number of embryos developed up to the stage of 2–4 blastomeres and of early blastula, in the presence of the test compounds, was evaluated using a microscope.

### 4.4. Ethical Approval for Human Blood Samples

The medical ethical committee of the local hospital (Vladivostok, Russian Federation) approved the study protocol. All subjects who participated in the experiments wrote an informed consent.

### 4.5. Leukocytes

Leukocytes were rapidly isolated from venous citrated blood by lyzing erythrocytes in a solution containing 0.15 M NH_4_Cl, 10 mM NaHCO_3_, and 0.1 mM EDTA [39].

### 4.6. Detection of Reactive Oxygen Production

Reactive oxygen production was detected by flow cytometry, with APF, as described elsewhere [40]. Cells (200,000 cells/well) were incubated for 1 hour, with the samples (12.5, 25, 50, and 100 µg mL^−1^; final value). Free cells with (phosphate buffer saline) PBS, instead of the samples, were used as the negative control and were considered to be 100%. After 10 min on ice, the cells were analyzed, immediately, by a four color FACSCalibur (Becton Dickinson, San Jose, CA, USA) flow cytometer. Forward and side scatter light was used to identify the neutrophils cell populations.

### 4.7. IL-6-, IL-10- and TNFα-Inducing Activity of CRGs and Ech on Human Blood Cells 

Blood processing was performed using the procedure described by De Groote et al. [41]. Heparinated peripheral blood was diluted 1:5 in sterile Medium 199 (Sigma, St. Louis, MO, USA) with glutamine (300 mg L^−1^) (Gibco, Darmstadt, Germany) and gentamicin (50 μg ml^−1^). Diluted blood (0.1 mL) was incubated with the investigated samples, in saline (37 °C, 5% CO_2_). The CRGs were added to obtain the final concentrations of 5, 10, and 20 μg mL^−1^ Ech (to 1 μg mL^−1^) and their complexes, to obtain final concentration values corresponding to the substances, separately. After 24 h, the supernatants were collected and frozen, followed by determining the cytokine content, using specific ELISA kits, according to the manufacture's protocol (“Cytokine”, Saint-Petersburg, Russia).

### 4.8. Cell culture

The human cancer cell line HT-29 was obtained from the American Type Culture Collection (https://www.lgcstandards-atcc.org/). HT-29 cells were incubated at 37 °C in a 5% CO_2_ humidified atmosphere, in McCoy’s 5a Medium Modified, containing 10% *v*/*v* FBS (Lot RWH35894, HyClone, Logan, UT, USA), 2 mM L-glutamine, and 1% penicillin/streptomycin (Invitrogen, Paisley, UK).

### 4.9. xCELLigence System

Experiments on the xCELLigence system were conducted by means of the Real-Time Cell Analyzer Dual Plate (RTCA-DP) instrument (ACEA Bioscience, San Diego, CA, USA). The recommendations proposed by Ke et al. [42] and Sokolova et al. [35] were applied to study the effect of the samples on human intestinal epithelial cell monolayers.

Samples were added as follows: 

Single samples. Carrageenans (20 μL, *C* = 250, 500, and 1000 μg mL^−1^); Ech (20 μL, *C* = 10 μg mL^−1^).

Complex samples. Carrageenan (10 μL, *C* = 500, 1000, and 2000 μg mL^−1^) + Ech (10 μL, *C* = 20 μg mL^−1^). (Concentrations are expressed as initial values.)

### 4.10. Statistical Analysis

All data are presented as means ± standard deviations. Statistical calculation was conducted by ANOVA one-way analysis of variance. Differences were suggested when statistically probable at *p* < 0.01.

## 5. Conclusions

The inclusion of Ech into the carrageenan matrix enables a decrease the spermotoxicity of the Ech (besides protection of the substance from oxidation and improvement its solubility [11]), preserving its protective properties against the ROS synthesis by neutrophils, and the protective action towards the HT-29 intestinal epithelial cells.

## Figures and Tables

**Table 1:** The structures of disaccharides repeating units of carrageenans from algae of Gigartinaceae and Phyllophoraceae families.

Reference	Algal Species	CRG Sample	Structures of Disaccharide Repeating Units		SO_4_^2^^−^ Contents
			3-Linked	4-Linked	
[20]	*C. armatus*	λ	G2S	D2S, 6S	26
[20]	*C. armatus*	κ	G4S	DA	22
[21]	*T. crinitus*	κ/β	G4S/G	DA/DA	19
[22]	*A. flabelliformis*	ι/κ	G4S/G4S	DA2S/DA	20
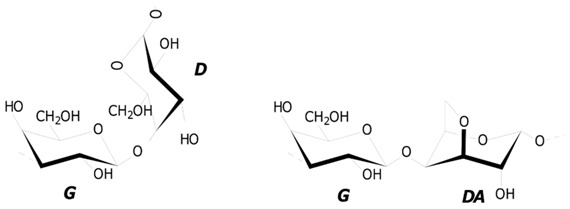					

## References

[1] Sumich J.L., Pinkard-Meier D.R. (2016). Introduction to the Biology of Marine Life.

[2] Pozharitskaya O.N., Shikov A.N., Laakso I., Seppänen-Laakso T., Makarenko I.E., Faustova N.M., Makarova M.N., Makarov V.G. (2015). Bioactivity and chemical characterization of gonads of green sea urchin *Strongylocentrotus droebachiensis* from Barents Sea. J. Funct. Foods.

[3] Jeong S.H., Kim H.K., Song I.S., Lee S.J., Ko K.S., Rhee B.D., Kim N., Mishchenko N.P., Fedoryev S.A., Stonik V.A. (2014). Echinochrome A Protects Mitochondrial Function in Cardiomyocytes against Cardiotoxic Drugs. Mar. Drugs.

[4] Pozharitskaya O.N., Ivanova S.A., Shikov A.N., Makarov V.G. (2013). Evaluation of free radical-scavenging activity of sea urchin pigments using HPTLC with post-chromatographic derivatization. Chromatographia.

[5] Brasseur L., Hennebert E., Fievez L., Caulier G., Bureau F., Tafforeau L., Flammang P., Gerbaux P., Eeckhaut I. (2017). The Roles of Spinochromes in Four Shallow Water Tropical Sea Urchins and Their Potential as Bioactive Pharmacological Agents. Mar. Drugs.

[6] Shikov A.N., Pozharitskaya O.N., Krishtopina A.S., Makarov V.G. (2018). Naphthoquinone pigments from sea urchins: Chemistry and pharmacology. Phytochem. Rev..

[7] Mishchenko N.P., Fedoreev S.A., Bagirova V.L. (2003). Histochrome: A new original domestic drug. Pharm. Chem. J..

[8] Elyakov G.B., Maximov O.B., Mischenko N.P., Koltsova E.A., Fedoreev S.A., Glebko L.I., Krasovskaya N.P., Artjukov A.A. (2007). Drug Preparation “histochrome” for Treating Acute Myocardial Infarction and Ischaemic Heart Diseases. European Patent.

[9] Santo V.E., Frias A.M., Carida M., Cancedda R., Gomes M.E., Mano J.F., Reis R.L. (2009). Carrageenan-based hydrogels for the controlled delivery of PDGF-BB in bone tissue engineering applications. Biomacromolecules.

[10] Shit S.C., Shah P.M. (2014). Edible polymers: Challenges and opportunities. J. Polym..

[11] Yermak I.M., Mischchenko N.P., Davydova V.N., Glazunov V.P., Tarbeeva D.V., Kravchenko A.O., Pimenova E.A., Sorokina I.V. (2017). Carrageenans-Sulfated Polysaccharides from Red Seaweeds as Matrices for the Inclusion of Echinochrome. Mar. Drugs.

[12] Knutsen S.H., Myslabodski D.E., Larsen B., Usov A.I. (1994). A modified system of nomenclature for red algal galactans. Bot. Mar..

[13] Falshaw R., Furneaux R. (1994). Carragenan from the tetrasporic stage of *Gigartina decipiens* (Gigartinaceae, Rhodophyta). Carbohydr. Res..

[14] Yermak I.M., Khotimchenko Y.S., Fingerman M., Nagabhushanam R. (2003). Chemical properties, biological activities and applications of carrageenan from red algae. Recent Advances in Marine Biotechnology.

[15] Pereira L., Soares F., Freitas A.C., Duarte A.C., Ribeiro-Claro P., Sudha P.N. (2017). Extraction, Characterization, and Use of Carrageenans. Industrial Applications of Marine Biopolymers.

[16] Pereira L. (2016). Edible Seaweeds of the World.

[17] Weiner M.L. (2014). Food additive carrageenan: Part II: A critical review of carrageenan in vivo safety studies. Crit. Rev. Toxicol..

[18] Zia K.M., Tabasum S., Nasif M., Sultan N., Aslam N., Noreen A., Zuber M. (2017). A review on synthesis, properties and applications of natural polymer based carrageenan blends and composites. Int. J. Biol. Macromol..

[19] Magnan S., Tota J.E., El-Zein M., Burchell A.N., Schiller J.T., Ferenczy A., Tellier P.-P., Coutlee F., Franco E.L. (2018). Efficacy of a Carrageenan gel Against Transmission of Cervical HPV (CATCH): Interim analysis of a randomized, double-blind, placebo-controlled, phase 2B trial. Clin. Microbiol. Infect..

[20] Yermak I.M., Kim Y.H., Titlyanov E.A., Isakov V.V., Solov’eva T.F. (1999). Chemical structure and gel properties of carrageenan from algae belonging to the Gigartinaceae and Tichocapaceae, collected from the Russian Pacific coast. J. Appl. Phycol..

[21] Barabanova A.O., Yermak I.M., Glazunov V.P., Isakov V.V., Titlyanov E.A., Solov’eva T.F. (2005). Comparative study of carrageenans from reproductive and sterile forms of *Tichocarpus crinitus* (Gmel.) Rupr (Rhodophyta, Tichocarpaceae). Biochem..

[22] Kravchenko A.O., Anastyuk S.D., Sokolova E.V., Isakov V.V., Glazunov V.P., Helbert W., Yermak I.M. (2016). Structural analysis and cytokine-induced activity of gelling sulfated polysaccharide from the cystocarpic plants of *Ahnfeltiopsis flabelliformis*. Carbohydr. Pol..

[23] Dinnel P.A., Link J.M., Sober Q.J. (1987). Improved methodology for a sea urchin sperm cell bioassay for marine waters. Arch. Environ. Contam. Toxicol..

[24] Lera S., Macchia S., Pellegrini D. (2006). Standardizing the methodology of sperm cell test with Paracentrotus lividus. Environ. Monit. Assess..

[25] Flannagan R.S., Jaumouillé V., Grinstein S. (2012). The cell biology of phagocytosis. Annu. Rev. Pathol. Mech. Dis..

[26] Dupré-Crochet S., Erard M., Nüβe O. (2013). ROS production in phagocytes: Why, when, and where?. J. Leukoc. Biol..

[27] Halliwell B., Whiteman M. (2004). Measuring reactive species and oxidative damage in vivo and in cell culture: How should you do it and what do the results mean?. Br. J. Pharmacol..

[28] Chan W.I., Zhang G., Li X., Leung C.H., Ma D.L., Dong L., Wang C. (2017). Carrageenan activates monocytes via type-specific binding with interleukin-8: An implication for design of immuno-active biomaterials. Biomater. Sci..

[29] Lennikov A., Kitaichi N., Noda K., Mizuuchi K., Ando R., Dong Z., Fukuhara J., Kinoshita S., Namba K., Ohno Sh., Ishida S. (2014). Amelioration of endotoxin-induced uveitis treated with the sea urchin pigment echinochrome in rats. Mol. Vis..

[30] Schieber M., Chandel N.S. (2014). ROS function in redox signaling and oxidative stress. Cur. Biol..

[31] Commins S.P., Borish L., Steinke J.W. (2010). Immunologic messenger molecules: Cytokines, interferons, and chemokines. J. Allergy Clin. Immunol..

[32] Inoue K., Takano H., Hiyoshi K., Ichinose T., Sadakane K., Yanagisawa R., Tomura S., Kumaga Y. (2006). Naphthoquinone enhances antigen-related airway inflammation in mice. Eur. Respir. J..

[33] McKim J.M., Baas H., Rice G.P., Willoughby J.A., Weiner M.L., Blakemore W. (2016). Effects of carrageenan on cell permeability, cytotoxicity, and cytokine gene expression in human intestinal and hepatic cell lines. Food Chem. Toxicol..

[34] Langerholc T., Maragkoudakis P.A., Wollgast J., Gradisnik L., Cencic A. (2011). Novel and established intestinal cell line models-an indispensable tool in food science and nutrition. Trends Food Sci. Technol..

[35] Sokolova E.V., Kuz’mich A.S., Byankina A.O., Yermak I.M. (2017). Effect of carrageenans alone and in combination with casein or lipopolysaccharide on human epithelial intestinal HT-29 cells. J. Biomed. Mater. Res. Part A.

[36] Vasileva E.A., Mishchenko N.P., Tran V.T.T., Vo H.M.N., Bui L.M., Denisenko V.A., Fedoreyev S.A. (2017). Quinoid pigments from the sea urchin *Astropyga radiata*. Chem. Nat. Compd..

[37] Buznikov G.A., Podmariov V.K., Dettlaff A., Vassetzky S.G. (1990). Sea urchins *Strongylocentrotus drobachiensis*, *S. Nudus*, *S. intermedius*. Animal Species for Developmental Studies.

[38] Pinsino A., Mantranga V., Trinchella F., Roccheri M.C. (2010). Sea urchin embryos as an in vivo model for the assessment of manganese toxicity: Developmental and stress response effects. Ecotoxicology.

[39] Lehmann A.K., Sornes S., Halstensen A. (2000). Phagocytosis: Measurement by flow cytometry. J. Immunol. Methods.

[40] Setsukinai K.I., Urano Y., Kakinuma K., Majima H.J., Nagano T. (2003). Development of novel fluorescence probes that can reliably detect reactive oxygen species and distinguish specific species. J. Biol. Chem..

[41] De Groote D., Zangerle P.F., Gevaert Y., Fassotte M.F., Beguin Y., Noizat-Pirenne F., Pirenne J., Gathy R., Lopez I.M., Dehart I. (1992). Direct stimulation of cytokines (IL-1β, TNF-α, IL-6, IL-2, IFN-γ and GM-CSF) in whole blood. I. Comparison with isolated PBMC stimulation. Cytokine.

[42] Ke N., Wang X., Xu X., Abassi Y.A. (2011). The xCELLigence system for real-time and label-free monitoring of cell viability. Methods Mol. Biol..

